# Employment of the Envisia Genomic Classifier in Conjunction With Cryobiopsy in Patients With Undiagnosed Interstitial Lung Disease

**DOI:** 10.1016/j.chpulm.2023.100034

**Published:** 2023-12-27

**Authors:** Fayez Kheir, Ramsy Abdelghani, Diana Espinoza, Regina Villalobos, David Becnel, Rachel Herr, Alejandro Aragaki, J.P. Uribe Becerra, Justin M. Oldham, Joseph Lasky

**Affiliations:** a Division of Pulmonary and Critical Care Medicine, Massachusetts General Hospital, Boston, MA; b Division of Pulmonary and Critical Care, Department of Medicine, School of Medicine, Tulane University, New Orleans, LA; c Division of Pulmonary, Critical Care and Sleep Medicine, University of Cincinnati Medical Center, Cincinnati, OH; d Department of Internal Medicine, University of Miami, Miami, FL; e Division of Pulmonary and Critical Care Medicine, University of Michigan, Ann Arbor, MI

**Keywords:** bronchoscopy, cryobiopsy, Envisia Genomic Classifier, interstitial lung diseases

## Abstract

**Background:**

The Envisia Genomic Classifier (EGC) is a clinically validated molecular diagnostic test identifying usual interstitial pneumonia (UIP), increasing the diagnostic confidence for idiopathic pulmonary fibrosis in patients with interstitial lung disease (ILD).

**Research Question:**

What is the association of the EGC and lung cryobiopsy on clinical management decisions in patients with fibrotic ILD?

**Study Design and Methods:**

Retrospective analysis of patients at multimedical centers. We assessed the change in management strategy after EGC and cryobiopsy. We evaluated the association between genomic UIP classification and disease progression using the American Thoracic Society definition of progressive pulmonary fibrosis (method 1), or combined end point of death from any cause, lung transplant, or ≥ 10% relative decline in FVC (method 2).

**Results:**

In patients that were EGC positive for UIP (gcUIP+), 78.6% were diagnosed with idiopathic pulmonary fibrosis. Cryobiopsy and EGC test results changed management strategy for 59.5% of patients in the cohort that were gcUIP+, and 21.4% of patients that had indeterminate cryobiopsy interpretations were gcUIP+, leading to a change in treatment strategy of an additional 16.7%. There was a decrease in follow-up without treatment from 64.3% to 11.9% (*P* < .001). Utilization of immunosuppressive drugs decreased from 23.8% to 9.5% (*P* = .06), and there was an increase in treatment with antifibrotics drugs from 11.9% to 71.4% (*P* < .001). A Kaplan-Meier curve of disease progression did not reach statistical significance on multivariable analysis (method 1: hazard ratio, 1.4; 95% CI, 0.4-4.2; *P* = .55; method 2: hazard ratio, 1.3; 95% CI, 0.8-2.1; *P* = .29). In analysis of EGC positivity for UIP, patients who were not prescribed antifibrotics showed disease progression compared with patients who were EGC negative for UIP (hazard ratio, 1.8; 95% CI, 0.99-3.4; *P* = .053)

**Interpretation:**

This study suggests that combined EGC and cryobiopsy are associated with change in therapeutic strategy in patients with undiagnosed ILD. The EGC might serve as a predictor for disease progression in patients not treated with antifibrotics.

**Take-home Points**

**Study Question:** What is the association of genomic classifier and lung cryobiopsy on clinical management decisions in patients with fibrotic interstitial lung disease?

**Results:** Genomic classifier and cryobiopsy results changed management strategy for patients with interstitial lung disease, and there was disease progression in patients with genomic classifier positive for usual interstitial pneumonia not on antifibrotics compared with patients with genomic classifier negative for usual interstitial pneumonia.

**Interpretation:** Combined genomic classifier and cryobiopsy is associated with change in therapeutic strategy in patients with undiagnosed interstitial lung disease, and genomic classifier might serve as a predictor for disease progression in patients not treated with antifibrotics.

Interstitial lung diseases (ILDs) include an array of complex and heterogeneous diseases that can be challenging to diagnose. A specific ILD diagnosis provides important prognostic and treatment implications. Idiopathic pulmonary fibrosis (IPF) is a progressive fibrotic ILD associated with high mortality.^1^ A pathology pattern of usual interstitial pneumonia (UIP) is what is observed in IPF, and an high-resolution CT (HRCT) scan pattern of UIP reported on a biopsy from other diseases (eg, rheumatoid arthritis) is associated with more rapid disease progression.^2^ Despite continued refinement of the updated IPF diagnostic guidelines, the accurate diagnosis of IPF remains challenging, often resulting in delayed or misdiagnosis that may lead to worse outcomes.^1^

In patients without a typical UIP CT scan pattern, current guidelines recommended bronchoscopic lung cryobiopsy (BLC) as an acceptable alternative to surgical lung biopsy (SLB) for making a histopathologic diagnosis.^1^ Although BLC is a relatively safe procedure with significantly lower morbidity and mortality compared with SLB, the diagnostic yield is only approximately 80%.^3^ The Envisia Genomic Classifier (EGC) (Veracyte), consisting of 190 genes previously identified through machine learning analysis of exon messenger RNA sequencing, is a commercially available diagnostic tool developed to predict histologic UIP in patients undergoing transbronchial biopsy.^4^ EGC testing predicts histopathologic UIP in patients with ILD with a specificity of 92% and sensitivity of 68%.^5^ Studies have demonstrated how the EGC may be incorporated into the diagnostic evaluation of patients with fibrotic ILD to increase diagnostic confidence in the absence of an SLB.^6^, ^7^, ^8^, ^9^, ^10^ Despite the EGC increasing diagnostic confidence, a critical remaining question is whether this test will impact management decisions potentially leading to quicker actionable diagnoses.

The aim of this study was to evaluate how the EGC together with BLC data is associated with a change of treatment strategy. We hypothesized that the addition of an EGC positive for UIP (gcUIP+) result to a BLC result would increase the proportion of patients prescribed antifibrotic therapy. We also explored the disease progression in patients with a gcUIP+ result compared with patients who were gcUIP- using two different disease progression definitions.

## Study Design and Methods

### Study Design and Patient Selection

This study was performed at 3 academic medical centers. A waiver of consent was provided by institutional review boards at each institution given the retrospective nature of the study. Informed consent for BLC and EGC was obtained from patients per institutional policy.

Consecutive patients aged ≥ 40 years with clinical and radiologic features of ILD, but without a definite UIP pattern on HRCT imaging per chest radiologist or experienced ILD pulmonologists, as defined by patterns described by Fleischner Society criteria^11^ pertaining to clinical practice guidelines who underwent both BLC and EGC from June 2019 to December 2022, were enrolled into this study. All patients were initially evaluated and followed-up by an ILD specialist or multidisciplinary team (MDT). Patients were followed for at least 18 months. The indication for BLC was based on a decision made by the ILD and interventional pulmonologists who evaluated the patients at each center. Patients without baseline spirometry and those lost to follow-up were excluded. During the MDT discussion after the addition of clinical, radiographic, and histologic information along with the EGC, the experts were asked to decide on case management (treatment or follow-up).

Disease progression was assessed using two methods. Method 1 was defined according to clinical practice guidelines^1^ as at least two of the following three criteria occurring within 1 year after bronchoscopy with no alternative explanation: (1) worsening respiratory symptoms, (2) physiologic evidence of disease progression (either of the following): absolute decline in FVC ≥ 5% predicted within 1 year of follow-up or absolute decline in diffusing capacity of the lung for carbon monoxide (Dlco) (corrected for hemoglobin) ≥ 10% predicted within 1 year of follow-up, or (3) radiologic evidence of disease progression. Method 2 has been used in prior clinical trials^12^ and is defined as the time from bronchoscopy to death from any cause, lung transplant, ≥ 10% relative FVC decline, or censoring. Patients were censored at 18 months or the date of last available FVC if performed < 18 months after bronchoscopy.

### Data Collection

A standard form was used to collect clinical information from electronic medical records including baseline demographics, HRCT pattern, medications including immunomodulatory agents and antifibrotics, histopathology and EGC data, and pulmonary function testing (FVC and Dlco). Longitudinal data obtained from the medical record included patient symptoms, serial lung function, HRCT imaging, and employment of immunomodulatory and antifibrotic medications.

### Organizational Scheme

The methodology described by Flaherty et al^13^ was used to evaluate the association of BLC and GC on clinical management decisions in patients with fibrotic ILD in the MDT process. The ILD cases were reviewed by the MDT which consisted of an ILD expert, chest radiologist, and pulmonary pathologist. The MDT reviewed clinical-radiologic findings, and diagnostics along with treatment plans were recorded in the medical records. The MDT then rediscussed cases once biopsy and EGC results were available.

### Study Outcomes

The primary objective of this study was to evaluate the association of combining BLC with the EGC result on patient treatment decisions in those that were gcUIP+ allowing a confident treatment strategy. A change in management was defined as a change in either treatment with antifibrotics or immunomodulatory medications, or follow-up decision (from follow-up to treatment or vice versa). The outcome measure was defined as a statistically significant and clinically meaningful (ie, in at least 20% of cases) change in management decision after consideration of biopsy and EGC findings. We also assessed the association of lung cryobiopsy on treatment decisions for patients that were EGC negative. Finally, we evaluated disease progression in patients who were gcUIP+ compared with patients who were gcUIP- using two definitions. We used method 1 on this cohort where data were available to assess progression. Also, we used method 2 combining data from our previously published study^14^ along with this cohort to assess progression.

### Bronchoscopic Lung Cryobiopsy

All transbronchial lung cryobiopsies were performed with a flexible bronchoscope by an experienced interventional pulmonologist, with the patient under general anesthesia. The biopsy site was chosen by the interventional bronchoscopist, taking into consideration the HRCT scan appearance of each patient. All cryobiopsies were performed under fluoroscopic guidance using a 1.9- or 1.7-mm diameter cryoprobe (ERBOKRYO; Erbe USA Inc).^15^^,^^16^ After assessing that the cryoprobe was about 1 cm from the pleura, the cryoprobe was activated for 5 to 8 s and then removed with bronchoscope en bloc in one quick motion. The entire process was repeated if the cryobiopsy was also obtained from a different lung segment. A Fogarty balloon occlusion catheter 5.0 (Edwards Lifesciences) was positioned at the entrance of the targeted segmental bronchus and used prophylactically after each cryobiopsy, or another bronchoscope was advanced into the airway and wedged at the bronchus to control bleeding.^14^^,^^15^ Biopsy from lower lung zones was prioritized if diffuse disease was seen on HRCT scan. In addition, lateral airways were preferred so that the cryoprobe intersects with the pleura in a perpendicular manner, allowing for improved visualization of probe to pleural distance on fluoroscopy. We aimed to attain two biopsies from two different areas of the same lobe to maximize yield.

### Statistical Analyses

Continuous variables are reported as mean ± SD, and categorical variables are reported as frequency (%). Normal distribution of all continuous variables was assessed using Shapiro-Wilk test and histograms. Comparisons were done using the *t* test or nonparametric Mann-Whitney U test as appropriate. Categorical variables were compared using the χ^2^ test. The McNemar test was used to compare the proportions of treatment before and after the genomic classifier test for the genomic UIP group. Time to disease progression is reported as Kaplan-Meier curves and were compared with the log-rank test. All tests were two-sided, with the significance level set to *P* < .05. Correction for multiple comparisons was performed using the Bonferroni method. All statistical analysis was performed using IBM SPSS Statistical software version 28 (IBM Corp) and R software version 4.3.0.

## Results

**Figure 1:**
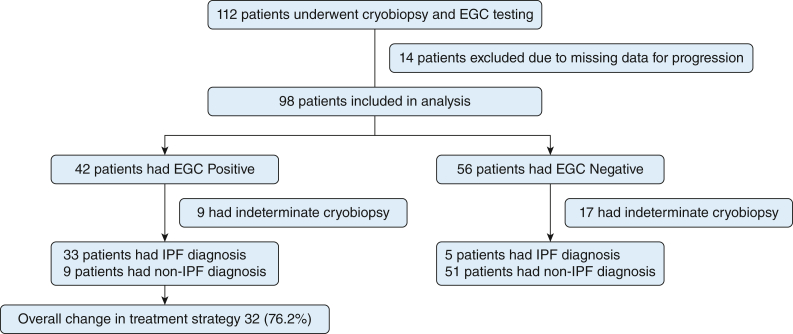
Algorithm showing the impact of cryobiopsy and genomic classifier information on patients’ management. EGC = Envisia Genomic Classifier; IPF = idiopathic pulmonary fibrosis.

A total of 112 patients underwent BLC and transbronchial biopsy for EGC testing between June 2019 and December 2022 (Fig 1). Among eligible patients, 14 were excluded because of missing data for disease progression. Of the 98 patients included in the final analysis, 42 (42.9%) were classified as gcUIP+ and 56 (57.1%) were classified as EGC negative. Patients with a gcUIP+ classification were older (Table 1). Baseline lung function, percentage of males, and smoking status were similar between groups. A higher proportion of those with a gcUIP+ classification were diagnosed with IPF (33 of 42, 78.6%), whereas a high proportion of those that were gcUIP- were diagnosed with non-IPF ILD (51 of 56, 91.1%) (Table 1). Among cases diagnosed with non-IPF ILD after EGC testing, unclassifiable ILD was most common, followed by idiopathic nonspecific interstitial pneumonia, fibrotic hypersensitivity pneumonitis, and connective tissue disease-associated ILD. Regarding baseline HRCT scan pattern, patients with a gcUIP+ classification had probable UIP (28.6%) and indeterminate (71.4%) radiologic patterns compared with patients that were gcUIP- who exhibited an HRCT scan pattern of probable UIP (10.7%), indeterminate (69.9%), and alternative (19.6%) patterns (Table 1).

**Table 1:** Patient Characteristics and Diagnoses Stratified by Genomic UIP Classification

Variable	EGC Positive (n = 42)	EGC Negative (n = 56)	*P* Value
Baseline characteristic			
Age, y	71 ± 8	61 ± 11	< .001
Male sex	28 (66.7)	32 (57.1)	.34
HRCT scan pattern			
Probable UIP	12 (28.6)	6 (10.7)	.02
Indeterminate UIP	30 (71.4)	39 (69.9)	.85
Alternative diagnosis	0 (0)	11 (19.6)	< .001
Pulmonary function			
FVC % predicted	72 ± 15	74 ± 16	.28
Dlco % predicted	52 ± 15	53 ± 16	.75
Patients with active tobacco use or who previously smoked	28 (66.7)	36 (64.3)	.81
Histopathology diagnosis			
UIP	26 (61.9)	8 (14.3)	< .001
Nonspecific interstitial pneumonia	1 (2.4)	13 (23.2)	< .001
Hypersensitivity pneumonitis	3 (7.1)	1 (1.8)	N/S
Interstitial fibrosis	9 (21.4)	21 (37.5)	.02
Other ILD	1 (2.4)	5 (8.9)	N/S
Nondiagnostic	2 (4.8)	5 (8.9)	N/S
Sarcoidosis	0 (0)	3 (5.4)	N/S
MDT diagnosis after cryobiopsy and EGC testing			
Idiopathic pulmonary fibrosis	33 (78.6)	5 (8.9)	< .001
Nonidiopathic pulmonary fibrosis	9 (21.4)	51 (91.1)	< .001
Fibrotic hypersensitivity pneumonitis	4 (44)	4 (7.8)	
Idiopathic nonspecific interstitial pneumonia	1 (11)	13 (25.5)	
Unclassifiable ILD	2 (22)	17 (33)	
Connective tissue disease-associated ILD	1 (11)	9 (17.6)	
Sarcoidosis	0 (0)	3 (5.9)	
Other ILD	1 (11)	5 (9.8)	

Values are mean ± SD, No. (%), or as otherwise indicated. Dlco = diffusing capacity of the lung for carbon monoxide; EGC = Envisia Genomic Classifier; HRCT = high resolution computed tomography; ILD = interstitial lung disease; MDT = multidisciplinary team; N/S = nonsignificant; UIP = usual interstitial pneumonia.

### Outcomes

#### Change in Treatment Strategy Before and After Combined Lung Cryobiopsy and Genomic Classifier Data

**Figure 2:**
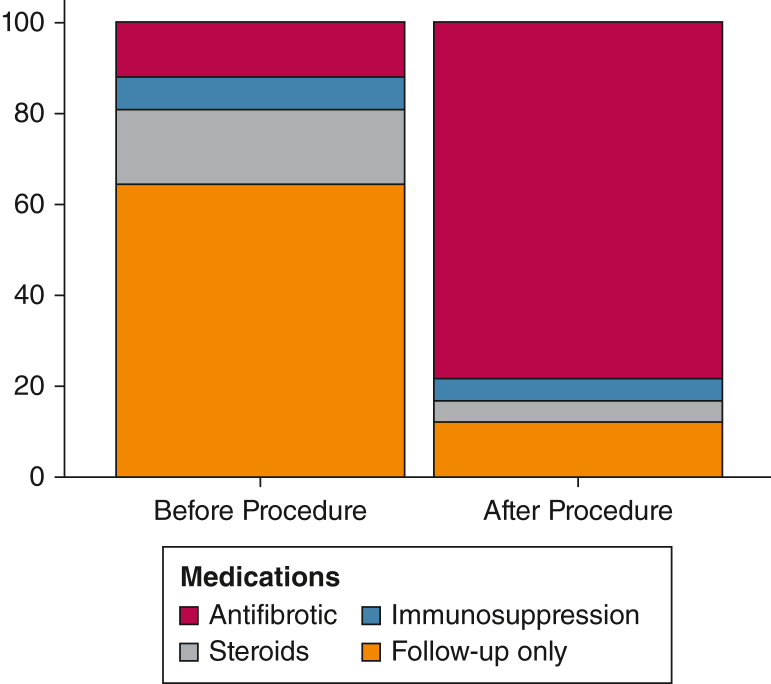
Bar plots that show the management plan in patients before and after the procedure (cryobiopsy and genomic testing).

Combined histopathologic and EGC data were associated with change in the treatment strategy for 25 of 42 patients (59.5%) in the gcUIP+ cohort. Nine patients out of 42 (21.4%) had indeterminate cryobiopsy interpretations with gcUIP+, eventually associated with change in treatment strategy of an additional 16.7% (seven of nine cases). Overall, the management strategy when combining BLC with EGC changed in 32 of 42 patients (76.2%) (Fig 2). There was a decrease in follow-up without treatment from 64.3% to 11.9% (*P* < .001) and use of immunosuppressive drugs from 23.8% to 9.5% (*P* = .06). Conversely, there was an increase in antifibrotic drug prescription from 11.9% to 71.4% (*P* < .001).

In the EGC-negative cohort, histopathologic data changed the treatment strategy for 39 of 56 patients (69.6%). There was a decrease in follow-up without treatment from 69.6% to 32.1% (*P* < .001), an increase in immunosuppressive drug prescription from 26.8% to 51.8% (*P* < .001), and an increase in prescriptions for antifibrotics drugs from 3.6% to 16.1% (*P* = .02).

### Disease Progression

**Figure 3:**
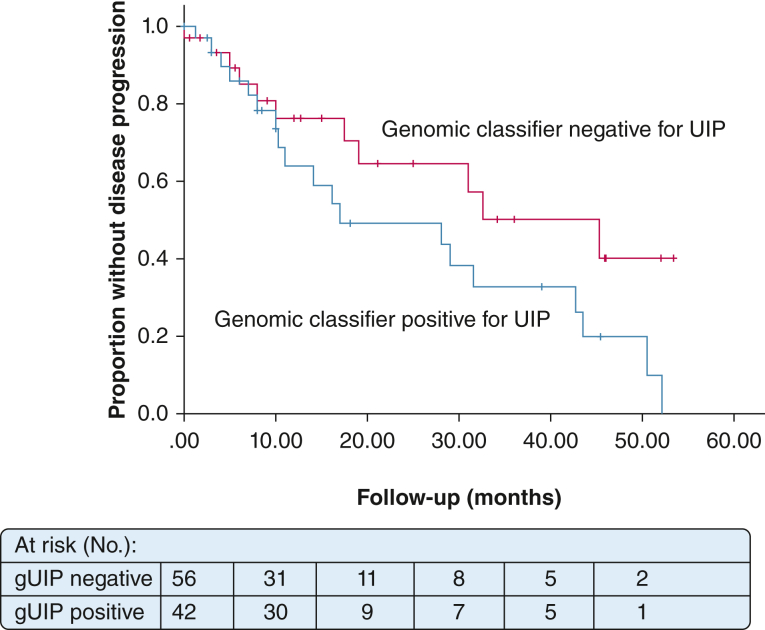
Kaplan-Meier plot for disease progression comparing patients with genomic classifier positive and negative for UIP pattern using method 1. gUIP = genomic usual interstitial pneumonia; UIP = usual interstitial pneumonia.

Kaplan-Meier plots for disease progression using method 1 are shown in Figure 3. We performed a multivariable Cox survival analysis adjusted by age, sex, smoking status, IPF diagnosis, radiologic pattern on HRCT scan, FVC % predicted, and Dlco. Kaplan-Meier analysis of disease progression according to gcUIP test results showed a clear separation of the gcUIP positive and negative curves, but this did not reach the statistical significance threshold (hazard ratio [HR], 1.4; 95% CI, 0.4-4.2; *P* = .55). Subanalysis of patients who were gcUIP+ who were not prescribed antifibrotics compared with patients who were gcUIP– did not show any statistical significance, possibly because of a small sample size.

**Figure 4:**
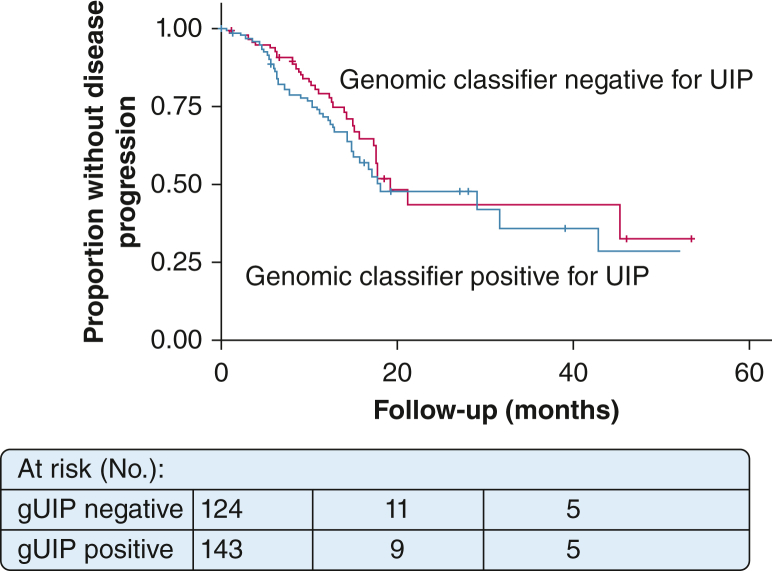
Kaplan-Meier plot for disease progression comparing patients with genomic classifier positive and negative for UIP pattern using method 2. gUIP = genomic usual interstitial pneumonia; UIP = usual interstitial pneumonia.

**Figure 5:**
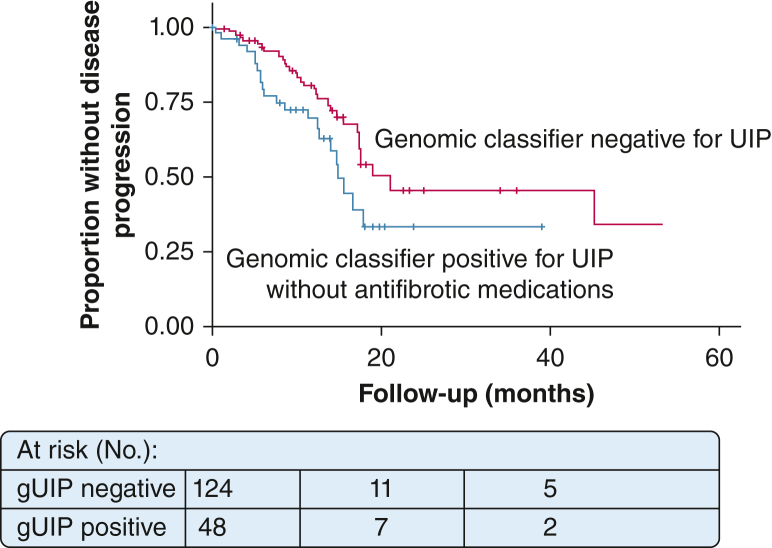
Kaplan-Meier plot for disease progression comparing patients with genomic classifier positive for UIP pattern without antifibrotic medications and negative for UIP pattern using method 2. gUIP = genomic usual interstitial pneumonia; UIP = usual interstitial pneumonia.

Kaplan-Meier plots for disease progression using method 2 are shown in Figure 4. We performed a multivariable Cox survival analysis adjusted by age, sex, smoking status, IPF diagnosis, radiologic pattern on HRCT scan, FVC % predicted, and Dlco. A gcUIP+ classification was associated with trend toward increased risk of disease progression (HR, 1.3; 95% CI, 0.8-2.1; *P* = .29) but did not meet the statistical significance threshold. Kaplan-Meier analysis of disease progression according to gcUIP test results did not reach the statistical significance threshold (HR, 1.3; 95% CI, 0.8-2.1; *P* = .29). Subanalysis of patients who were EGC positive who were not prescribed antifibrotics showed disease progression compared with patients with EGC-negative UIP classification (HR, 1.8; 95% CI, 0.99-3.4; *P* = .053) (Fig 5).

## Discussion

Previous studies have demonstrated that a positive EGC strongly associates with a pathologic UIP pattern and may help increase the confidence in a diagnosis of IPF.^5^, ^6^, ^7^, ^8^ However, there is discussion of a paradigm shift with establishing a UIP pattern as a single, separate diagnostic entity.^2^^,^^17^ This is because secondary fibrotic ILD (eg, autoimmune or hypersensitivity pneumonitis) with a definite UIP HRCT scan pattern share similar disease progression to IPF with major prognostic implications.^12^ Therefore, the question faced by many physicians managing patients with ILD is moving from diagnosis to prognosis and a management-focused approach.

A US-based pulmonologist clinical decision-making survey evaluated the impact of the EGC on clinical diagnosis and antifibrotic recommendation of cases with fibrotic ILD without a typical UIP pattern on HRCT scan.^10^ There was a 36% increase in antifibrotic recommendation in the pre-/post-EGC cohort when information was given in a staged fashion and 11% increase in antifibrotic recommendation in an independent cohort when all information was given simultaneously. The EGC can predict histopathologic UIP in patients with ILD with a 92% specificity but only 68% sensitivity,^3^ indicating that histopathology may be required for a confident diagnosis in the subset of patients who were EGC negative. Our study represents a real-world application of EGC decision-making for patients with fibrotic ILD. Overall, BLC was indeterminate in 26.5% of patients (26 of 98) with fibrotic ILD, which aligns with published literature.^3^ Adding the EGC to BLC resulted in an additional nine patients that were gcUIP+ where BLC was indeterminate. Therefore, combined EGC and histopathologic data were associated with change of therapeutic strategy in 59.5% of patients in our study, and this increased to 76.2% when the EGC was positive for UIP and the BLC was indeterminate. This 16.7% increase in management decision could potentially increase appropriate antifibrotic prescriptions and reduce the risk of initiating harmful immunosuppressive therapies in patients with IPF.^18^

Chaudhary et al^14^ compared progression-free survival defined as composite outcomes (time to death, lung transplant, or FVC decline ≥ 10% up to 18 months) in a retrospective, multicenter, US-based cohort study between patients that were EGC positive vs EGC negative for UIP. This study did not show a statistically significant association between disease progression and an EGC positive pattern. However, the study was limited by a small sample size and relatively short-term follow-up, indicating the need for larger-scale studies investigating the utility of EGC and prognosis in such patients. Also, similar to this study, treatment of patients who were EGC positive with antifibrotics may have slowed disease progression.

The study assessed disease progression as a secondary outcome using the current American Thoracic Society/European Respiratory Society/Japanese Respiratory Society/Latin American Thoracic Society guidelines^1^ definition (method 1) and the definition used in the Chaudhary et al^14^ paper (method 2). Both methods did not show a statistically significant difference in disease progression in patients that were gcUIP+ compared with gcUIP-. However, there was a trend toward statistical significance when patients who were EGC positive not treated with antifibrotics were compared with patients with EGC-negative results. This may in part be because the study was not adequately powered to show statistical difference. Also, we had a relatively short follow-up and a high proportion of patients who were gcUIP+ were started on antifibrotic medications. However, our results should be interpreted with caution because 16% of patients with gcUIP-negative results were prescribed antifibrotics. Although our study supports the need for larger prospective trials to evaluate the prognostic and therapeutic implications of a positive UIP EGC result, this may not be ethical. However, unlike Chaudhary et al,^14^ our study showed that all patients with an alternate diagnosis HRCT scan pattern were EGC negative for UIP and therefore the GC may be less likely to influence treatment management in such cases. The discrepancy between the two studies regarding this point may be related to reported differences in HRCT interobserver classification agreement. The final diagnoses of patients who were EGC negative in the cohort with alternative HRCT scan interpretations included sarcoidosis (n = 3), connective tissue disease-associated ILD (n = 4), nonspecific interstitial pneumonia (n = 1), smoking-related interstitial fibrosis (n = 2), and cryptogenic organizing pneumonia (n = 1). These results should be viewed with caution because the number of patients with alternate diagnosis pattern on HRCT scan was small (n = 11). Furthermore, in the appropriate clinical setting, biopsy may not be needed for patients with a radiographic pattern of probable UIP if the clinical context suggests IPF. However, some patients with such a pattern may have abnormal, but nonspecific, autoantibodies, a significant environmental or work exposure history, or medication regimens that are associated with ILD, and therefore may require a biopsy to establish whether they have IPF or an alternative diagnosis. What is more, there is a nontrivial element of discordance in HRCT scan interpretations for ILD.^19^

This study has some limitations. First, this study was performed in academic referral centers for ILDs with expertise in BLC and may not be applicable to other community-based centers or global centers where BLC is not yet used. Second, a total of 19.4% of cases were viewed as unclassifiable, which is comparable with the percentage of unclassifiable cases despite MDTs in patients undergoing SLB.^17^ Third, as previously discussed, the sample size and follow-up were relatively small, and larger studies will be helpful to corroborate our findings. Fourth, it was not possible to ascertain whether pathologists or radiologists were blinded to clinical data because this study reflected real-world daily clinical practice where charts are available to physicians if needed. Fifth, this study assessed the combined assessment of the EGC and BLC, without the ability to look at the effects of each of these two components alone. However, the impact of sequentially presented data from BLC and the EGC on the diagnostic confidence of the MDT was previously published.^8^ Finally, this is a retrospective study with all inherent biases stemming from that and can only show association between combining EGC and BLC data with respect to change in treatment strategy. Further prospective clinical trials are needed to assess such causality.

## Interpretation

This study demonstrated that combining BLC and the EGC was associated with change in treatment strategy leading to a decrease in follow-up only or employment of immunosuppressive medications in favor of antifibrotic prescription in patients with a UIP EGC pattern. The EGC might serve as a biomarker for progression in patients with fibrotic ILD.

## Funding/Support

The authors have reported to *CHEST Pulmonary* that no funding was received for this study.

## Financial/Nonfinancial Disclosures

Fayez Kheir reports a relationship with Veracyte, Biodesix and UpToDate that includes: speaking and lecture fees. Ramsy Abdelghani receives consulting fees from intuitive.Joseph Lasky reports a relationship with Veracyte and Boehringer Ingelheim that includes: speaking and lecture fees. Joseph Lasky reports a relationship with Galecto, United Therapeutics and Genentech that includes: consulting or advisory. Justin M Oldham reports a relationship with Boehringer Ingelheim, Lupin Pharmaceuticals, AmMax Bio, Roche and Veracyte that includes: speaking and lecture fees. Justin M Oldham reports a relationship with Endeavor Biomedicines that includes: consulting or advisory. Justin M Oldham has patent issued to US20200155494A1. None declared (D.E.,R.V.,D.B.,R.H.,A.A.,J.U.B.)
